# Emotional-Behavioral Regulation, Temperament and Parent–Child Interactions Are Associated with Dopamine Transporter Allelic Polymorphism in Early Childhood: A Pilot Study

**DOI:** 10.3390/ijerph17228564

**Published:** 2020-11-18

**Authors:** Silvia Cimino, Eleonora Marzilli, Mimma Tafà, Luca Cerniglia

**Affiliations:** 1 Department of Dynamic and Clinical Psychology, University of Rome, 00136 Rome, Italy; silvia.cimino@uniroma1.it (S.C.); eleonora.marzilli@uniroma1.it (E.M.); mimma.tafa@uniroma1.it (M.T.); 2 Faculty of Psychology, International Telematic University Uninettuno, Corso Vittorio Emanuele II, 39, 00136 Rome, Italy

**Keywords:** dopamine transporter, genotype, psychopathological risk, temperament, feeding interactions

## Abstract

International research has highlighted the role played by individual genetic polymorphism, children’s emotional-behavioral functioning, and quality of parent–child feeding interaction in shaping children’s development. Few studies have focused on the dopamine transporter (DAT1) gene in these processes. In a community sample of 81 families with young children aged between 19 and 28 months (37 males and 44 females), this pilot study aimed to explore possible relationships between children’s DAT1 genotype (9/x: 9/9, 9/19 contrasted to 10/10), their own psychological profiles, parental psychopathological risk, and the quality of mother–child and father–child feeding interactions. Children’s DAT1 genotype was assessed collecting DNA through buccal; children’s temperament and emotional-behavioral regulation, and parental psychopathological risk were assessed, respectively, through report-form and self-report instruments; then, dyadic exchanges were videotaped during a mealtime, and coded based on the Scala di Valutazione dell′Interazione Alimentare (SVIA). Results showed significant differences in the variables under study based on children’s DAT1 genotype, with a higher risk associated with the 10/10. Our findings have provided preliminary new evidence on the relationship between a different child’s polymorphisms, their own emotional-behavioral functioning, and the quality of the family environment, with important implications for the planning of more targeted prevention programs.

## 1. Introduction

Recent international research in the field of developmental psychopathology has shown that the key to a better understanding of children’s emotional-behavioral functioning is paying attention to the mutual influences between multiple levels of analysis [1,2]. Clinicians and researchers within an ecological-transactional perspective of development [3] have identified specific risk domains among early childhood, including parental characteristics (i.e., personality characteristics, psychopathological risk, etc.) [4,5,6,7,8], the quality of the parent–child relationship (e.g., attachment, dyadic interactions) [9,10,11,12,13], the child’s individual characteristics (e.g., temperament, cognitive abilities, emotional-behavioral functioning, etc.) [14,15,16], and the inherited genetic features (i.e., polymorphic and/or epigenetic variations within specific genes) [17,18,19,20,21]. In this field, it is widely shown that children’s and parent’s psychopathological difficulties may mutually influence each other, with bidirectional effects [22,23,24]. Moreover, a large body of research has demonstrated that the quality of parent-child exchanges may be negatively influenced by parental psychopathology and/or children’s temperament and psychopathological symptoms, leading to dyadic interaction characterized by scarce involvement, high prevalence of negative affective states, and interactive conflict [25,26,27]. Children’s genetic features may in turn affect these relationships, lead to a higher risk of children’s internalizing and externalizing problems [17,18,19,20,21].

Genes influencing the dopaminergic system are studied in this field due to their crucial involvement in the regulation of numerous processes central to the child’s emotional-adaptive and behavioral functioning, such as reward-motivated behavior [28], mood [29], attention and aggression [30,31]. The release of dopamine (DA) is also associated with motivational aspects functional to exploration [32] and affiliation behaviors [33], as well as in reward-motivated processes involved in the regulation of food intake [34,35] and in the hedonic aspects of feeding [36,37]. The availability of DA in the brain is modulated primarily by its transporter (a protein known as active dopamine transporter (DAT)), which recaps DA at the level of nerve synapses, and whose level of activity is regulated by the expression of its gene (DAT1) [38]. Consequently, it constitutes the most important control mechanism of extracellular DA [39,40]. In humans, DAT1 (also known as SLC6A3) is located in the chromosomal region 5p15.3 [41]. The 3 ’untranslated region (3′-UTR) of the DAT1 gene contains a variable number of tandem repetitions (VNTRs) of 40 polymorphic base pairs (bp), near the polyadenylation site. The sequence can be repeated 3–11 times, but several studies have shown that it occurs with highest frequency in the 9- and 10-repeat forms [42,43]. Importantly, the different genotypes may affect the DAT1 expression and the subsequent phenotypic variation [44,45,46].

A dysregulated DAT activity, in terms of its excessive or inhibited functionality, could lead to an imbalance in intracellular DA levels [47], promoting non-adaptive behaviors as a result of a chemical imbalance in the brain [48,49]. This imbalance plays a crucial role in an individual’s disease susceptibility, and studies on clinical samples have shown that DAT1 was associated with different psychopathological difficulties, from infancy to adulthood, including attention-deficit hyperactivity disorder (ADHD) [50,51,52,53], conduct disorder [54], affective disorders [55,56], post-traumatic stress disorder [57], and eating disorders [58,59]. A couple of studies have focused on the general population, showing significant associations with children’s temperament traits (including uninhibited traits, negative emotionality, aggressive and impulsive traits) [60,61,62], symptoms of ADHD [63,64], and other subclinical forms of psychological difficulties in both internalizing and externalizing areas [65,66,67,68]. However, to date, only a few studies have focused on early childhood [61,62], and to our best knowledge, no studies have explored the possible association between children’s DAT1 polymorphism with children’s dysregulation profile (DP), an empirically-based profile of poor self-regulation among early childhood [69], with which prospective associations with psychopathological difficulties have been evidenced over time [70,71,72].

Moreover, previous studies have reported conflicting results regarding which is the DAT1 polymorphic variation most associated with children’s psychopathological difficulties [51,66,73,74]. Recent studies suggest that DAT1 genotypes could have a different effect on children’s adjustment based on the specific development phase: the 10/10 genotype would be associated with a greater risk in childhood, while the 9/x genotype with a higher risk in adulthood [75].

Research has also shown the presence of significant associations between children’s DAT1 genotype with risk factors of family environment, emphasizing the role played by parental psychopathological symptoms [67,68], and a poor quality of mother–child relationship (mainly maternal–filial relationship) [61,76,77]. Indeed, intrinsic (genetic) and extrinsic (environmental) influences are not necessarily independent factors; in some cases, a genetic variation can also influence how the child modifies or creates their own environmental experiences [78], a phenomenon defined gene-environment correlation (rGE) [79]. Specifically, in the case of evocative rGEs, specific genetically influenced characteristics and behaviors of the child (e.g., temperament traits, emotional-behavioral functioning) tend to elicit certain types of response from the environment (e.g., the quality of their parental–filial interactions) [80,81]. However, to date, although many studies have highlighted the key role assumed also by the quality of the mother–child relationship in shaping children’s emotional-behavioral development [8,9,10,13], no studies have yet explored possible associations of the quality of father-child interactions with children’s DAT1 genotype. Moreover, the quality of parent–child interactions during feeding has not yet been evaluated, although the research has amply highlighted the central role played by DA in the regulation on feeding behaviors [34,35].

Based on these premises and literature gaps, this pilot study aimed to verify the presence of possible relationships between children’s DAT1 genotype and their own psychological profiles, parental psychopathological risks, and the quality of parent–child dyadic interactions among a community sample of families with young children. In particular, we aimed to verify whether: (a) children with 9/x polymorphism showed different scores of emotional-behavioral regulation, temperament, and internalizing/externalizing symptoms if compared to 10/10 children; (b) verify whether the quality of their interactions with their parents significantly differed.

## 2. Materials and Methods

### 2.1. Participants

The sample consisted of *N* = 107 children and their parents (mothers and fathers) recruited thanks to the collaboration of public and private kindergartens in the Center of Italy. Parents were contacted by psychologists who explained the aims of the study. Families who agreed to participate signed a written informed consent in which the steps of the study were detailed. The study was approved by the Ethical Committee of the Psychology Faculty at Sapienza University of Rome (Protocol Number 27/2016), following the Declaration of Helsinki.

The inclusion criteria of the sample were: the age range of children from 18 to 36 months; the absence of physical or mental disorders in parents and/or children; both parents taking care of the feeding of the child in the absence of their partner; both parents being biological parents of the child. We excluded families in which children and/or parents were following a pharmacological or psychological treatment (*N* = 4); families in which the children had physical or psychological disabilities (*N* = 6), or in which parents reported current or previous psychological disorders (*N* = 4); families in which parents did not complete all the questionnaires (*N* = 9); and families in which mothers or fathers were not the biological parents of the child (*N* = 3).

Therefore, the final sample of the present study was composed of *N* = 81 families with children aged between 19 and 28 months (37 males and 44 females), with an average age of 23.4 months (ds = 2.01). The average age of the mothers was 31.73 (ds = 1.87) and that of the fathers was 36.94 (ds = 2.97). All parents were Caucasian and most of them had high school or university (89%) education; 94% of couples were married and all families had average socioeconomic status (93% had an average income of 25,000–30,000 euros per year).

Based on the children’s DAT1 genotype (children with at least one 9-repeat allele and those with two copies of 10-repeat allele) the sample was divided into two groups: (1) 9/*x* group, composed by families in which the child carried a 9-repeat allele (9/9 or 9/10) of the DAT1 gene (*N* = 40); (2) 10/10 group, families in which the child carried a 10/10 DAT1 genotype (*N* = 41).

### 2.2. Procedure

Children’s biological samples were collected at kindergartens through buccal swabs (Isohelix Swab Pack) from which it is possible to extract the DNA present in the epithelial cells and subsequently examine gene expression of DAT1. Parents were previously informed that, for at least 1 h before collecting the child’s salivary sample, they should not have eaten (including chewing gum, sweets, etc.), drunken (exception water) or brushed their teeth. Once the buccal swabs were collected, they were slightly chilled by normal ice (+4 °C). After the administration of tampons, parents filled the *Symptom Checklist-90-Revised* (SCL-90-R) [82,83], a self-report for the assessment of their own psychopathological symptoms, and two report-form instruments, the *Questionari Italiani del Temperamento* (QUIT) [84], for the assessment of children’s temperament, and the *Child Behavior Checklist* (CBCL 1 ½–5) [85,86] for the assessment of children’s emotional-behavioral functioning. The two parents filled out separately and independently the CBCL 1 ½–5 and the QUIT; given that mothers and fathers reports obtained an interrater agreement respectively of 91% and 93%, we used mean scores. Moreover, parents were asked to interact with their children during the main meal, in the home, on two different days. Mother–child and father–child feeding interactions were videotaped (20 min videos), based on a validated procedure [87,88]. The observational data were then coded by two independent raters (Cohen’s k = 0.84), trained to the use of the instrument in normal and clinical populations, at the “Sapienza”, University of Rome. For reliability, the average agreement with expert encoders was between 0.83 and 0.97. This tool was chosen because is the only observational procedure for the assessment of both mother–child and father–child interactions during feeding validated for the Italian population.

### 2.3. Measures

The QUIT [84] is a parent-questionnaire validated in an Italian sample to assess the child’s temperament from the first month to 11 years of age, within 4 age groups: from 1 to 12 months, 12 to 36 months, 3 to 6 years, and 7 to 11 years. It is composed of 60 items to investigate six dimensions of the child’s temperament: 1) level of motor activity, (2) attention capacity, (3) inhibition to novelty, (4) social orientation (readiness for socialization), (5) positive emotionality, and (6) negative emotionality. The questionnaire shows good internal consistency (Cronbach’s α = 0.59–0.71).

The CBCL 1½–5 [85,86] is a report–form questionnaire 99-item informant-report questionnaire for the assessment of emotional/behavioral problems of the child. Parents are asked to rate 99-items, which are grouped in two broad-band scales: internalizing problems (which combines the scores of emotionally reactive, anxious/depressed, somatic complaints, withdrawn syndrome scales) and externalizing problems (comprised of scores of attention problems and aggressive behavior syndrome scales). This tool is one of the most validated instruments used to assess childhood emotional and behavioral problems [89,90,91]. For the assessment of CBCL DP scores, the items of the syndrome scales anxious/depressed, attention problems, and aggressive behavior were summed [92].

The SCL-90-R [82,83] is a 90-item self-report questionnaire aimed to assess psychological symptoms and psychological distress in adults. This instrument is widely used for screening and assessment of psychological symptoms in adults of both clinical and the general population [93,94,95,96,97,98]. Items are measured on a Likert scale ranging from 0 (not at all) to 4 (extremely). It is composed of nine primary dimensions (somatization, obsessive-compulsivity, interpersonal sensitivity, depression, anxiety, hostility, phobic anxiety, paranoid ideation, and psychoticism), but it is possible to calculate a global severity index (GSI) to provide the severity and degree of psychological distress, used for the aim of this study. The Italian validation [83] showed good reliability in terms of internal consistency (Cronbach’s α = 0.70–0.96).

The Scala di Valutazione dell′Interazione Alimentare (SVIA) [88] is the Italian adaptation of the Feeding Scale [87] that can be applied to children 12–36 months old. It measures interactive behaviors and identifies normal and/or risky relational modes between a parent and child during feeding exchanges. Parent–infant interactions during feeding are recorded for at least 20 min, and then a wide range of interactive mother–infant behaviors are coded and evaluated. The SVIA consists of 41 items distributed among four subscales: (1) Parent’s affective states (index of the parent’s affective states); (2) Interactive conflict (index of interactions characterized by conflictual, non-collaborative, and non-empathetic communication); (3) Food refusal behavior (habits associated with challenged status regulation during meals and with limited food consumption); and (4) Dyad’s affective state (index of the extent to which the infant’s feeding patterns are, or are not, the result of an interactive regulation to which both partners contribute). Higher scores on each scale refer to greater difficulties. The SVIA showed a good reliability in terms of internal consistency (Cronbach’s α = 0.79–0.96).

### 2.4. DNA Isolation and Genotyping

DNA extraction from the buccal wall cells was performed using the Buccal-Prep Plus DNA isolation (Isohelix), following the manufacturer’s instructions. The DAT1 polymorphism was determined by amplifying the repeated sequence of the 3 ’untranslated (3′-UTR) region, by the polymerase chain reaction (PCR) technique [51,53]. Based on previous studies in the field of developmental psychopathology, which compared children with at least one allele 9- repeated (9/x; 9/9, 9/10) [53,61,67,68], results will be reported considering the presence/absence of the 9/x allele (10/10 vs. 9/9, 9/10).

### 2.5. Statistical Analyses

Preliminary analyses were performed using descriptive statistics (frequencies, percentages, and mean scores). One-way analyses of variance (ANOVAs) and Chi-square tests were used to examine possible differences between the two groups in terms of family members’ age and children’s gender, and to identify potential confounding variables. Based on preliminary results, in the main analyses, children’s age was included as a covariate. To verify significant differences between the two groups on children’s temperament and emotional-behavioral functioning, two multivariate analysis of covariance (MANCOVA) tests were conducted using, respectively, the scales of QUIT (i.e., social orientation; inhibition to novelty; level of motor activity; positive emotionality; negative emotionality; attention capacity) and of CBCL 1.5-5 (i.e., internalizing problems, externalizing problems, and DP) as dependent variables, and the Group as “factor”. Both models included children’s age, maternal and paternal GSI as covariates. Then, to verify possible differences in maternal and paternal psychopathological risk between the study groups, ANCOVAs were carried out, separately for mothers and fathers, with the group as the independent variable, mother’s and father’s scores of GSI/SCL-90-R as dependent variables. Children’s age, and the scores of QUIT and CBCL 1.5-5 were included as covariates. Finally, the differences between the two groups on the quality of mother–child and father–child feeding interactions were examined using two MANCOVA, with the group as the independent variable and each of the four subscales and of the SVIA as dependent variables. Mothers’ and fathers’ scores of GSI, children’s age, and the scales of QUIT and CBCL 1.5-5 were included as covariates. For all main analyses, partial eta squared (ηp^2^) were reported as a measure of effect size in terms of the partial variance explained by the independent variable. All analyses were performed using IBM SPSS Statistics software, Version 25.0 [99].

## 3. Results

### 3.1. Preliminary Analyses

Results of ANOVAs showed that children’s age, but not parental age, was significantly different in the two groups; children with the 10/10 DAT1 genotype were older than children with the 9/x genotype (Table 1). Consequently, children’s age was included as a covariate in the main analyses. Chi-square analyses showed no significant differences between the groups on children’s gender.

### 3.2. Children’s Temperament and Emotional-Behavioral Functioning in the Two Groups

As regards child’s temperament, results of the MANCOVA test showed no significant covariate effects, but there was a significant effect of group (*λ* = 0.22, *F* (6.71) = 537.01, *p* = 0.000, *η*_p_^2^ = 0.97). The univariate effects, revealed that children with 10/10 genotype had significantly lower scores of inhibition to novelty, positive emotionality, and attention capacity than children of the 9/x genotype group. Consequently, the scores of these dimensions of QUIT were included as a covariate in the subsequent analyses (Table 2).

Regarding children’s emotional-behavioral functioning, MANCOVA showed that maternal GSI was a significant covariate (*λ* = 0.89, *F*(3.74) = 2.82, *p* = 0.04, *η*_p_^2^ = 0.10), and after controlling for this, children’s DAT1 genotype had a significant effect on the scores of CBCL 1.5-5 scales (*λ* = 0.02, *F*(3.74) = 1048.92, *p* = 0.000, *η*_p_^2^ = 0.97). In the subsequent univariate analyses, we included maternal GSI as a covariate, because it was a significant predictor in the MANCOVA. Results of ANCOVAs indicated that children’s with 10/10 genotype had higher scores than children with a 9/x genotype on the scales of internalizing and externalizing problems, and on CBCL DP (Table 3). The maternal GSI covariate effect did not prove statistically significant for the analyses of Internalizing and externalizing problems. However, for the analyses of DP, maternal GSI exerted a significant covariate effect (*F*(1.78) = 4.13, *p* = 0.04, *η*_p_^2^ = 0.05).

### 3.3. Maternal and Paternal Psychopathological Risk in the Two Groups

As regards maternal psychopathological risk, the ANCOVA showed that the main effect of group was not significant (*F*(1.72) = 0.15, *p* = 0.94, *η*_p_^2^ = 0.00), but there were significant covariate effects of children’s positive emotionality (*F*(1.72) = 4.64, *p* = 0.03, *η*_p_^2^ = 0.060 and DP (*F*(1.74) = 4.50, *p* = 0.03, *η*_p_^2^ = 0.05). Regarding paternal psychopathological risk, there were no significant main or covariate effects (Table 4).

### 3.4. Quality of Mother–Child and Father–Child Feeding Interactions in the Two Groups

As regards the quality of mother–child feeding interactions, results indicated a significant effect of Group (*λ* = 0.83, *F*(4.68) = 3.42, *p* = 0.01, *η*_p_^2^ = 0.16), and a statistically significant covariate effect for maternal GSI (*λ* = 0.86, *F*(4.68) = 2.84, *p* = 0.03, *η*_p_^2^ = 0.13). The univariate analyses, controlling for maternal GSI, indicated that mother-child dyads of 10/10 group had higher scores in the SVIA subscale of affective state of the mother, food refusal of the child, and affective state of the dyad compared to dyads of the 9/x Group. The maternal GSI covariate effect was statistically significant only for the analyses of interactional conflict (*F*(1.78) = 8.77, *p* = 0.004, *η*_p_^2^ = 0.10).

Regarding the quality of father–child interactions, results of the MANCOVA showed a significant main effect of group (*λ* = 0.33, *F*(4.68) = 35.73, *p* = 0.000, *η*_p_^2^ = 0.66), and a significant covariate effect for children’s internalizing problems (*λ* = 0.87, *F*(4.68) = 2.53, *p* = 0.04, *η*_p_^2^ = 0.12). The univariate analyses, controlling for children’s internalizing problems, showed that father–child dyads of the 10/10 group showed higher scores on interactional conflict, food refusal of the child, and affective state of the dyad than in the other groups. Children’s internalizing problems had a statistically significantly covariate effect only for the analyses of food refusal of the child (*F*(1.78) = 6.27, *p* = 0.01, *η*_p_^2^ = 0.07) (Table 5).

## 4. Discussion

The aim of this pilot study was to investigate, in a community sample of families with young children, offspring emotional-behavioral functioning and temperament, psychopathological risk of their parents, and the quality of parent–children feeding interactions (with both mothers and fathers, separately), considering the role played by children’s DAT1 polymorphism.

Although the bidirectional influence between children’s and parental psychological profiles, as well as their impact on the quality of parent–children interaction is widely shown [22,23,24,25,26,27], a growing body of research has recently evidenced the key role played by individual genetic disposition and its dynamic interplay with the environment in shaping children’s evolutionary trajectory. We decided to consider the dopaminergic system as it is central to the regulation of the emotional-behavioral functioning of children [28,29,30,31,32,33], and in reward-motivation mechanisms related to feeding behaviors [34,35].

Furthermore, rather than concentrating only on the clinical population, recent evidence has suggested implementing research on families in community samples with children in their first years of life. Indeed, during sensitive periods of development, often psychopathological difficulties tend to occur subthreshold, leading to maladjustment of children in their living environment [100].

Overall, our results confirmed that children with a last one 9-repeat allele (9/x) showed significantly different scores from children carrying the 10/10 genotype in the variables under study, with a higher children’s psychopathological risk and poorer quality of feeding interactions associated with the latter. Specifically, children with 10/10 polymorphism showed higher uninhibited temperament, lower attention capacity, and express lower positive emotions than children with a 9/x genotype. Moreover, they had higher levels of psychopathological difficulties, in all considered areas (i.e., internalizing and externalizing problems, and emotional-behavioral dysregulation) compared to their peer with a 9/x polymorphism. Children’s DAT1 genotype explained for a large amount of variance of children’s temperament and of emotional-behavioral functioning (i.e., more than 90% of the variance). Interestingly, maternal psychopathological risk exerted a significant covariate effect on children’s emotional-behavioral dysregulation problems, but accounting for a lower percentage of variance compared to those explained by children’s 10/10 genotype (i.e., 5% vs. 62%). These findings are in accordance with previous studies that have shown that children who show an uninhibited temperament, poor attention capacity, and little positive emotionality also manifest emotional-behavioral self-regulation difficulties [101,102,103]. However, our study has added to previous literature new evidence to the fact that the DA path could be involved in the underpinning mechanism shared by these difficulties [62], suggesting a higher risk in the presence of 10/10 DAT1 genotype. Since the pioneering study by Cook and coll. [73] significant associations between 10/10 DAT1 genotype and children’s psychopathological problems (i.e., ADHD) have been found. Subsequently, many studies have replicated the same association for children’s other psychopathological difficulties [60,61,62,63,64,65,66,67,68], as also our study confirmed. However, to our knowledge, this is the first study to show that children’s DAT1 polymorphism can significantly correlate with emotional-behavioral dysregulation among early childhood. Furthermore, the studies by Fuke and coll. [44] and by Pastinen and coll. [45] have suggested that DAT1 polymorphisms can alter the functionality and availability of DA in the brain. Consequently, it could be hypothesized that the worst developmental outcomes associated with the 10/10 genotype, also depend on the expression levels of the DAT protein and the consequent DA imbalance [46]. Indeed, some studies have shown that DAT1 10/10 genotype was associated with greater gene expression of DAT [44,104] and consequent excessive removal of DA, compared to the 9/x genotype [105,106]. However, other studies have shown conflicting results, reporting either a density of DAT associated with the genotype 9/x [107] or no effect associated with individual genotype [108].

The inconsistent findings of genetic research supported the importance of considering the possible interplays between children’s genetic disposition and environment, especially family context, for a better understanding of vulnerability and resilience in early childhood [1,18]. Our preliminary results showed that, considering the role played by children’s psychopathological risk, children’s DAT1 genotype did not exert main effects on parental psychopathological risk. However, there were a significant covariate effects of children’s temperament (i.e., positive emotionality) and emotional-behavioral dysregulation problems on maternal psychopathological risk, which explained respectively 6% and 5% of the variance. These findings supported the evidence that children’s and maternal psychopathological risks may have a bidirectional and mutual influence on each other [22,23,24], beyond the possible children’s genetic influences. However, the greatest risk we found in the presence of the 10/10 genotype seems to have also been reflected a poorest quality of dyadic feeding interactions, with both mothers and fathers. Specifically, mother-child dyadic interactions of the 10/10 genotype group were characterized by the expression of a poorer affective state (both of the dyad and of the mothers), and children exhibited higher refusal behaviors compared to the dyads of the 9/x genotype group. Although maternal psychopathological risk had a significant covariate effect on the quality of dyadic interaction during feeding, the effect size was smaller than that exerted by children’s DAT1 genotype (i.e., 13% vs. 16% of the explained variance). Interestingly, maternal psychopathological risk leads to a higher interactional conflict during feeding, accounting for 7% of the variance, whereas children’s DAT1 genotype did not show an effect on this specific dimension of feeding interactions. Conversely, the quality of father–child feeding interactions was not significantly different between the two groups in the scores of affective states of the father. However, father–child dyads of the 10/10 genotype group showed lower levels of dyadic affective state and higher levels of interactional conflict than dyads of the other group. Although children’s internalizing problems had a significant covariate effect on the quality of father–child feeding interaction, it accounted for a smaller amount of variance compared to children’s 10/10 genotype (i.e., 16% vs. 66% of the variance). Moreover, the post-hoc univariate analyses of covariance showed that higher children’s internalizing problems are associated with higher levels of affective state of the fathers. These findings may suggest that children’s psychopathological risk may be directly related to fathers’ psychopathological risk, whereas children’s genetic influence would tend to exert its effects especially on the relational characteristics of the interactions. Our study may support the theoretical and empirical literature showing that mothers and fathers contribute in a distinct and peculiar way in interactions with their children [9,109,110].

In line with the research in the field of rGE [61,79,80,81], our study suggested that children’s DAT1 genotype may influence the quality of feeding interactions predisposing the child to specific temperamental characteristics, emotional-behavioral functioning, and food refusal behaviors, which in turn would tend to evoke a poorer quality of parental behaviors during the dyadic exchanges. Notably, children’s emotional and behavioral difficulties that we found associated with a 10/10 genotype (i.e., internalizing problems and dysregulation profile) are associated with maternal psychopathological risks, that in turn are significantly related to the quality of mother-child feeding interactions. Conversely, although we did not find a significant association between fathers’ psychopathological risk and those children’s psychopathological problems that were associated with children’s DAT1 genotype, we found a significant effect played by children’s internalizing problems on the quality of affective state expressed by fathers during the exchanges with their children.

This pilot study has some limitations that should be considered when interpreting its findings. Foremost, the study population consisted of a small sample size compared to the standards of genetic studies, which limits the generalizability of the results and lead to limited statistical power. Further investigations with a larger samples, providing more statistical power are, therefore, needed to confirm these preliminary results. Moreover, although we have assessed children’s and parental psychopathological risk through report-form and self-report instruments widely validated and extensively used in this field, our preliminary findings on the relationship between genetic and emotional-behavioral functioning may reflect the parental perception of their children, and should be taken with caution. Nevertheless, the use of an observational procedure of parent–child interaction has allowed us to have a more objective measure of parents’ and children’s behaviors, but further studies should use more robust instruments for the assessment of the emotional-behavioral functioning of family members (e.g., clinical interview). In addition, recent research in the field of gene–environment interaction has highlighted the role played by epigenetic mechanisms (i.e., methylation of DNA) in altering gene expression [111] and the consequences on individual behavior in response to environmental exposure [112]. Although some studies on clinical and non-clinical populations of children and adults [53,67,68,113] have evidenced the link existing between DAT1 methylation and psychopathological risk of parents and children, further studies in this field should be focused on the quality of the parent–child interactions during feeding. Furthermore, the genetic characteristics of mothers and fathers have not been evaluated, although the literature has shown that the DAT1 genotype of parents can in turn influence the quality of parent–children relationship and the consequences on children’s adjustment. Finally, given the cross-sectional design of this study and the relatively small sample recruited, it is not possible to define causal conclusions from our findings. As evidenced above, to verify our preliminary findings future research using a larger sample, and within a longitudinal study, is important.

Notwithstanding the above limitations, the present pilot study has several strengths. First, to our knowledge, no other study has considered the possible relationship between children’s DAT1 genotype and father–child interactions, although several studies have shown the importance of the quality of paternal exchanges for children’s development and that children’s genetic characteristics can moderate these relationships [54,68,77]. Moreover, despite the evidence of the involvement of DA and DAT1 genotypes in the regulation of feeding behaviors and eating disorders [34,35,36,37,58,59], this is the first study that has addressed possible associations between DAT1 with the quality of parent–child interactions during feeding. Finally, we used an observational and naturalistic validated tools to study parent–infant feeding interactions during feeding, which has allowed for an objective measure of the family members’ emotional-behavioral functioning. In this regard, some authors have recently underlined the utility of cross-sectional studies involving observation measures because their findings can be informative for the planning of appropriate prevention and treatment programs.

## 5. Conclusions

This pilot study has provided new preliminary evidence on the relationship between different child’s polymorphisms, their own emotional-behavioral functioning, and the quality of the family environment, showing the presence of specific characteristics associated with children’s DAT1 genotype. Our results have supported the presence of a higher risk associated with 10/10 genotype in early childhood, both in terms of higher psychopathological difficulties that a lower quality of parent–children interactions, that in turn are considered a crucial risk factor for children’s psychological well-being. Overall, further longitudinal research with larger study populations are needed to provide higher statistical power and support our preliminary findings, in order to promote the planning of more targeted programs for child development. Specifically, our preliminary findings could be informative for the early identification of children at higher risk for psychopathology due to their genetic influence (i.e., children with 10/10 genotype), that are potentially evocative of those environmental risks commonly associated with children’s internalizing and externalizing problems (i.e., the quality of parent–child interactions). Moreover, if specific parental qualities (i.e., the quality of dyadic exchanges, the presence of maternal psychopathological risk) had a mediating role on the relationship between children’s genotype and emotional-behavioral functioning, preventive interventions may be potentially targeted on the change of these environmental factors, to make parents less responsive to those children’s genetic behavioral influences. Further studies should confirm the presence of children’s behavior strongly influenced by DAT1 genotype. These children may need integrated multidisciplinary treatments, including psychosocial, behavioral, and pharmacological interventions. In this context, as also our findings have shown, it is important to note that the influence of environment on children’s psychopathological difficulties is not driven exclusively by children’s DAT1 genotype (and behaviors related to it), but may be influenced by a series of factors not necessarily related to children’s genetic features. However, given that environmental influences may interact with genetic influence in complex ways, beyond rGE processes, these dynamic mechanisms (i.e., gene–environment interaction, epigenetic processes) should be taken into account in further research, to improve the planning of increasingly individualized interventions based on both genetic and environmental vulnerabilities.

## Figures and Tables

**Table 1:** Analysis of variance (ANOVA) and Chi-square analyses results.

	Children’s DAT1 Genotype			
	9/x	10/10		
	M (SD)	M (SD)	F_1.79_	*p-*Value
Children’s age	22.63 (1.86)	24.15 (1.93)	13.01	0.001 **
Mothers’ age	31.83 (1.86)	31.63 (1.89)	0.20	0.64
Fathers’ age	36.48 (2.00)	37.39 (2.81)	2.84	0.09
Children’s Gender	***N* (%)**	***N* (%)**	***χ*^2^_2_**	***p*-Value**
Female	22 (55)	22 (53.7)		
Male	18 (45)	19 (46.3)	0.01	0.90

Note. ** *p* < 0.001.

**Table 2:** Univariate results of the differences between the two groups on children’s temperament.

Children’s DAT1 Genotype					
QUIT	9/x M (SD)	10/10 M (SD)	F_1.79_	*p*-Value	*η*_p_^2^
Social Orientation	4.09 (0.11)	4.10 (0.09)	0.53	0.46	0.007
Inhibition to Novelty	2.94 (0.88)	2.87 (0.11)	8.89	0.004 *	0.10
Level of Motor Activity	2.96 (0.06)	2.93 (0.09)	3.78	0.06	0.04
Positive Emotionality	3.82 (0.16)	2.28 (0.18)	1551.09	0.000 ***	0.95
Negative Emotionality	2.23 (0.08)	2.19 (0.07)	3.80	0.06	0.04
Attention Capacity	3.75 (0.12)	2.37 (0.16)	1901.01	0.000 ***	0.96

Note. *η*_p_^2^ = partial eta-squared. * *p* < 0.05, *** *p* < 0.001. QUIT: Questionari Italiani del Temperamento.

**Table 3:** Univariate results of the differences between the two groups on children’s emotional-behavioral functioning, controlling for maternal psychopathological risk.

Children’s DAT1 Genotype					
CBCL 1.5–5	9/x M (SD)	10/10 M (SD)	F_1.78_	*p*-Value	*η*_p_^2^
Internalizing Problems	10.50 (1.15)	27.51 (2.45)	1420.74	0.000 ***	0.94
Externalizing Problems	10.48 (1.18)	21.72 (2.33)	688.25	0.000 ***	0.89
Dysregulation Profile	13.94 (1.85)	28.34 (6.85)	132.10	0.000 ***	0.62

Note. *η*_p_^2^ = partial eta-squared. *** *p* < 0.001. CBCL: Child Behavior Check-List.

**Table 4:** Univariate results of the differences between the two groups on parental psychopathological risk, controlling for children’s age, temperament and emotional-behavioral functioning.

Children’s DAT1 Genotype					
SCL-90-R	9/x M (SD)	10/10 M (SD)	F_1.72_	*p*-Value	*η*_p_^2^
GSI of mothers	0.18 (0.09)	0.35 (0.30)	0.15	0.94	0.05
GSI of fathers	0.29 (0.26)	0.33 (0.30)	1.60	0.21	0.02

Note. *η*_p_^2^ = partial eta-squared. GSI = global severity index. SCL: Symptom Check-List.

**Table 5:** Univariate results of the differences between the two groups on mother–child feeding interactions (controlling for maternal psychopathological risk) and father–child feeding interactions (controlling for children’s internalizing problems).

Children’s DAT1 Genotype					
SVIA	9/x M (SD)	10/10 M (SD)	F_1.78_	*p*-Value	*η*_p_^2^
Mothers					
Affective State of the Mother	3.64 (0.51)	6.76 (0.86)	338.20	0.000 ***	0.81
Interactional Conflict	3.49 (0.48)	3.48 (0.46)	1.38	0.24	0.01
Food Refusal of the Child	1.86 (0.19)	1.69 (0.27)	13.26	0.000 ***	0.14
Affective State of the Dyad	2.21 (0.54)	3.82 (0.33)	221.45	0.000 ***	0.74
Fathers					
Affective State of the Father	7.07 (0.61)	7.29 (0.57)	0.42	0.51	0.005
Interactional Conflict	6.32 (0.49)	14.49 (0.77)	174.56	0.000 ***	0.69
Food Refusal of the Child	3.44 (0.47)	3.38 (0.52)	6.57	0.01	0.07
Affective State of the Dyad	3.41 (0.40)	8.34 (0.74)	66.92	0.000 ***	0.46

Note. *η*_p_^2^ = partial eta-squared. *** *p* < 0.001. SVIA: Scala di Valutazione delle Interazioni Alimentari.
